# TB programme stakeholder views on lessons from the COVID-19 response in South Africa

**DOI:** 10.5588/pha.23.0015

**Published:** 2023-09-21

**Authors:** H. Myburgh, S-A. Meehan, D. T. Wademan, M. Osman, A. C. Hesseling, G. Hoddinott

**Affiliations:** 1 Desmond Tutu TB Centre, Department of Paediatrics and Child Health, Faculty of Medicine and Health Sciences, Stellenbosch University, Cape Town, South Africa;; 2 Amsterdam Institute for Social Science Research (AISSR), University of Amsterdam, Amsterdam; Netherlands; 3 Amsterdam Institute for Global Health & Development, Amsterdam, Netherlands;; 4 School of Human Sciences, Faculty of Education, Health & Human Sciences, University of Greenwich, London, UK

**Keywords:** TB control, political will, public health

## Abstract

**BACKGROUND:**

The global COVID-19 pandemic has reversed many of the hard-won gains made in TB programmes and the associated reduction in the number of TB deaths, case notifications and incidence over the last three decades. Modelling estimates show that the impact will be lasting. There are global calls to recover the shortfalls along the TB care cascade that have resulted from COVID-19, with the recognition that the COVID-19 response holds lessons to inform more robust and comprehensive TB programmes and services.

**OBJECTIVE:**

To explore lessons from response measures to the COVID-19 pandemic in two high TB burden South African provinces.

**DESIGN:**

This was an exploratory qualitative study. We conducted interviews with TB programme stakeholders (managers and facility-level staff: *n* = 35) between February and June 2022.

**RESULTS:**

We identified eight facilitators of the COVID-19 response, including political will, rapid policy development, multi-sectoral collaboration, patient-centred models of care delivery, community engagement, mHealth and telehealth technologies, rigorous contact tracing and widespread mask wearing. Political will was singled out as a critical driver of the response.

**CONCLUSION:**

Leveraging COVID-19 inspired collaborations, technologies and avenues for health service delivery is an opportunity to maximise benefits for the TB programme. Reinvestment in national TB programmes and political prioritisation of TB are critical.

## Abstract (fr)

**CONTEXTE:**

La pandémie mondiale de COVID-19 a réduit à néant une grande partie des gains durement acquis dans les programmes de lutte contre la TB et la réduction associée du nombre de décès dus à la TB, de notifications de cas et d’incidence au cours des trois dernières décennies. Les estimations de la modélisation montrent que l’impact sera durable. Des appels ont été lancés au niveau mondial pour combler les lacunes dans la chaîne de soins de la TB qui ont résulté de la pandémie de COVID-19, en reconnaissant que la réponse à cette pandémie est porteuse d’enseignements qui permettront d’élaborer des programmes et des services de lutte contre la TB plus solides et plus complets.

**OBJECTIF:**

Etudier les enseignements tirés des mesures prises en réponse à la pandémie de COVID-19 dans deux provinces sud-africaines à forte charge de morbidité TB.

**MÉTHODE:**

Il s’agit d’une étude qualitative exploratoire. Nous avons mené des entretiens avec les parties prenantes des programmes de lutte contre la TB (responsables et personnel au niveau des établissements : *n* = 35) entre février et juin 2022.

**RÉSULTATS:**

Nous avons identifié huit facilitateurs de la riposte au COVID-19, notamment la volonté politique, l’élaboration rapide de directives, la collaboration multisectorielle, les modèles de prestation de soins centrés sur le patient, l’engagement communautaire, les technologies de mHealth et de télésanté, la recherche rigoureuse des contacts et le port généralisé de masques. La volonté politique a été désignée comme un moteur essentiel de la riposte.

**CONCLUSION:**

L’exploitation des collaborations, des technologies et des moyens inspirés du COVID-19 pour la prestation de services de santé est une occasion de maximiser les avantages pour le programme de lutte contre la TB. Il est essentiel de réinvestir dans les programmes nationaux de lutte contre la TB et d’en faire une priorité politique.

TB remains one of the leading causes of death globally from a single infectious agent with an estimated 1.6 million deaths reported in 2021, up from 1.4 million in 2019.^1^ The global COVID-19 pandemic and response – the height of which was experienced in 2020 and 2021 – have reversed many of the hard-won gains made in global TB programmes and associated reductions in the number of TB deaths, case notifications and incidence over the last three decades.^2^,^3^ Modelling estimates show that the impact will be lasting.^4^ There are global calls to recover the shortfalls along the TB care cascade that have resulted from COVID-19, with the recognition that the COVID-19 response holds lessons to inform more robust and comprehensive TB programmes and services going forward.^5^–^8^

South Africa is classified as a high TB burden country in all three of the WHO classifications – TB, TB-HIV and drug-resistant TB; it is one of only 10 countries included in all three of these classifications.^9^ It also reported the highest COVID-19 caseload on the African continent.^10^ In addition to these significant disease burdens, South Africa has the largest number of people living with HIV in the world and high communicable and non-communicable disease comorbidity.^11^ The anticipated interactions between TB, HIV, COVID-19 and non-communicable diseases led the country to implement a comprehensive COVID-19 response that was lauded internationally.^12^ The response involved stakeholders at all levels of government and the health system and sparked massive innovation and collaboration in a short space of time. The responses highlighted potential opportunities for strengthening TB control in South Africa through proposed health systems strengthening initiatives. These developments aim to enhance the overall capacity of the health system and hold promise for improving TB control efforts in the country in the future.^13^

We aimed to explore TB programme managers’ and frontline TB service providers’ perspectives on lessons from the COVID-19 response that can be applied to the TB programme and services in future. Specifically, we describe 1) factors that participants identified as facilitating the COVID-19 response, and 2) how these could be transferred to South African TB programming.

South Africa’s high TB burden has its roots in the country’s colonial and apartheid past which created ‘the social, economic, and environmental conditions […] for effective transmission’.^14^ The health system under apartheid was similarly grossly inadequate to meet the health needs of the majority of the population, and actively undermined effective TB prevention, diagnosis and treatment.^15^,^16^ In this context, the global HIV epidemic that emerged in the 80s and early 90s, just as South Africa was transitioning to its first democratically elected government, found fertile ground for transmission and fuelled the existing TB epidemic.^17^ While the HIV epidemic brought significant international and domestic health systems investment and medical innovations into South Africa, similar investments in TB were constrained both globally and locally.^18^

South Africa’s first ever national Tuberculosis Prevalence Survey, reporting on 2018 data, found a high TB prevalence in the population overall (737 per 100 000 population), and significant sub-clinical TB (57.8% bacteriologically confirmed, with no TB symptoms).^19^ The survey also found severe delays with care-seeking, with nearly two-thirds of those with symptoms not having sought care at the time the survey was conducted. These statistics were reported prior to the onset of the COVID-19 pandemic in 2020, which saw an unprecedented response from South Africa, including complete nationwide lockdown and mandatory mask wearing for all persons in public spaces. Alert Levels were implemented, reduced and reinstated in response to COVID-19 infection rates between March 2020 and April 2022, with significant and variable restrictions on movement.^20^,^21^ These circumstances negatively impacted TB care-seeking, diagnosis, treatment and outcomes.^12^,^22^,^23^ The large-scale public health response to COVID-19 in South Africa potentially holds lessons for the TB programme’s recovery, which we explore in this paper.

## METHODS

### Study design

This was an exploratory qualitative study.

### Setting

This study involved one-off semi-structured interviews with TB programme stakeholders in two South African provinces of KwaZulu-Natal (KZN) and Western Cape (WC) between February and June 2022. South Africa’s KZN and WC Provinces are two of the country’s three highest TB burden provinces, with reported incidence rates of ∼680/100 000 population.^24^ By September 2022, each province reported more than 700 000 confirmed COVID-19 cases (719 000 in KZN and 704 000 in the WC) since the first South African COVID-19 case was detected in March 2020 – the second and third highest COVID-19 caseloads in the country.^25^

We conducted interviews 2 years after the first COVID-19 cases appeared in South Africa. At the time, the South African government had reduced COVID-19 regulations to Adjusted Alert Level 1 – the lowest since the pandemic began – which involved mandatory mask wearing and isolation for persons testing positive for COVID-19 with symptoms. In April 2022, the National State of Disaster that had been in place since March 2020 was lifted, removing all COVID-19-related restrictions and mandates.

### Sampling

Working across the two provinces facilitated diversity in demographic profiles,^26^ health governance and service delivery structures, and urban/rural sprawl. In each province, with close collaboration with the Departments of Health, we 1) identified provincial and district-level participants involved in the TB programme to invite to the study, and 2) purposively selected six primary health facilities from one health district in each province from which to invite facility-level participants. Facilities were selected to ensure a mix of high and low TB caseloads. Provincial and district-level participants responded to email and telephonic invitations to participate in the study. At each of the 12 primary health facilities we invited two to three staff members involved in delivering TB services to participate in the study. Participants included doctors, nurses, community health workers, counsellors and data capturers.

### Data collection

Researchers conducted interviews with participants in person, telephonically or via virtual platforms; most interviews with facility-level participants were conducted in person at the health facilities. Researchers used a discussion guide to facilitate interviews, which included open-ended questions about participants’ involvement in the COVID-19 response, how the response unfolded in their place of work, changes to the TB programme during COVID-19, the processes for screening, testing and contact tracing for TB and COVID-19, adherence support for TB patients during COVID-19, mHealth technologies used during COVID-19, and community perceptions about TB and COVID-19. We specifically asked participants to reflect on lessons from the COVID-19 response for the TB programme. Where possible, we conducted interviews in participants’ language of choice (English, Afrikaans or Zulu). Interviews were between 25 min and 1 h in duration and were audio-recorded.

### Analysis

Detailed case descriptions were generated from audio recordings of interviews using a study-specific template. A deductive and inductive coding strategy was applied to the case descriptions to identify themes by participant type using ATLAS.ti software (ATLAS.ti Scientific Software Development, Technical University, Berlin, Germany) (Table). These preliminary findings were shared with project team members in the form of a summary report and workshopped to develop and sense-check interpretation.

**TABLE:** Deductive and inductive coding strategy applied to interview case descriptions

Deductive (pre-defined) codes	Inductive codes
Community challenges COVID-19 impact on TB service/programme Actions to support TB patients/service during COVID-19 Contact management/tracing Screening/testing mHealth technologies Mask wearing Key lesson	Challenges specific to TB Challenges specific to people ­affected by TB Community concern about TB Fear and uncertainty Health/TB programme involvement in COVID-19 response Multi-sectoral involvement Rapid policy development Integrated data systems Routine data publicly available Patient-centred care Telehealth/call centre Political will and commitment Poor TB programme implementation TB stigma Community engagement

### Ethical considerations

The study received ethics approval from Stellenbosch University Health Research Ethics Committee (Tygerberg), as well as provincial approval for the research to be conducted from the KZN (Pietermaritzburg) and WC provincial (Cape Town, South Africa) Departments of Health. Written informed consent was provided by all participants, including permission for the interview to be audio-recorded and for the researcher to take notes of the discussion. Participants were given study numbers to maintain confidentiality, and we indicate only participant type and which province they served when reporting findings to maintain participant anonymity.

## RESULTS

### Participant overview and involvement in the COVID-19 response

We interviewed 11 manager participants from multiple health districts and 24 facility-level participants across the 12 selected health facilities in the WC and KZN. Manager participants were involved in the HIV/AIDS, STIs and TB (HAST) or TB programmes at the district or provincial level, and were directly involved in coordinating and supervising the COVID-19 response in their respective places of work. Manager participants could easily reflect on the COVID-19 response and potential lessons for the TB programme. Facility-level participants reported limited involvement in delivering the COVID-19 response, with primary involvement in preparing their facilities for COVID-19 infection prevention control. Facility-level participants more easily recounted the impact on service delivery. They also shared community and patient-level challenges they experienced in delivering TB services.

### Facilitators of a fast and comprehensive COVID-19 response

We identified eight facilitators of the speed and comprehensiveness of the COVID-19 response in South Africa (Figure). These included political will, rapid policy development, multi-sectoral collaboration, mHealth and telehealth technologies, rigorous contact tracing, patient-centred models of care delivery, community engagement and widespread mask wearing (see Supplementary Data A for illustrative quotes from interviews).

**FIGURE:**
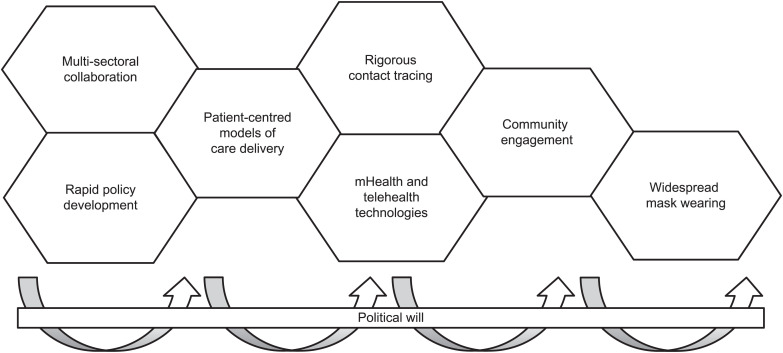
Facilitators of the speed and comprehensiveness of South Africa’s COVID-19 response. The Figure depicts the eight facilitators of the speed and comprehensiveness of the COVID-19 response in South Africa. It illustrates political will as the key driver and enabler of each of the facilitators identified and of the response overall.

### Political will

Participants singled out political will and focus as the catalyst and critical driver of South Africa’s COVID-19 response.

We had the Health Department and the political principals pulling in the same way…you didn’t have so much civil society kind of motivation, but you certainly had strong political commitment (Manager, March 2022, WC).

Another manager shared that,

COVID has forced our political leaders to be much more active in the health space, and to appreciate … how crucial the health system is within the bigger societal system (Manager, March 2022, WC).

Participants experienced the strong political will and commitment as enabling policy development processes, multi-sectoral collaboration, rigorous contact tracing, mHealth and telehealth innovations, community engagement and population-level mask wearing:

It’s the focus that actually made so much of a difference with regards to the COVID [response] being so successful in the tracing and testing of patients… You find that because the entire district was watching, everybody had to do what they needed to do (Manager, March 2022, KZN).

Participants were keenly aware that the international nature of the COVID-19 pandemic raised and retained the political attention of the South African government. Even as COVID-19 numbers had significantly declined in 2022 at the time of the interviews, participants said that critical health services resources were still being diverted away from the TB programme, to prioritise implementation of COVID-19 vaccinations, for example.

### Rapid policy development

Strong political will manifested in what participants perceived to be high-quality policies being developed and released rapidly, demonstrating that a faster, more responsive process of policy development is possible. A manager participant explained that ‘policies were passed overnight. Literally,’ and that they learnt that ‘by being more responsive [in their work] you sometimes have a better impact than by having the perfect answer’ (Manager, March 2022, WC). Participants pointed out that this rapid policy development process was facilitated by the collaboration with public health specialists from both the Department of Health and academic institutions, along with the mindset that ‘we need to get the job done with urgency’ (Manager, March 2022, WC). The same manager lamented that, ‘we’ve lost the urgency for TB and HIV’. However, another manager was optimistic, saying that ‘people are now awake…more people are now complaining about the speed [at which things get done for TB]’ (Manager, March 2022, WC/KZN).

### Massive multi-sectoral collaboration

Participants described how the massive multi-sectoral collaboration in the COVID-19 response played a pivotal role in enabling the response to unfold swiftly and effectively, with a heightened sense of urgency and scale. This included public–private partnerships within the health sector, between government departments, with universities and private businesses, as well as more flexible and responsive financial support from implementing partners. These collaborations were instrumental in enabling massive public health communication (posters, leaflets, TV, radio, billboards, social media), otherwise inaccessible in infection hotspots like schools and workplaces, for screening, testing and vaccination. ‘[I’ve] never seen [it] before, that we use everyone’s skills’, a manager explained, referring to the collaboration between non-profit organisations, academics, and health services (February 2022, WC).

Other managers commented on the public–private health sector partnerships, which allowed integrated data systems that had been an unrealised aspiration in health services.

### Patient-centred models of care delivery

Some managers reported that the COVID-19 response was patient-centred care in practice, with a focus on fostering patient autonomy, decision-making and trust; a lot of thought went into how to ‘reduce the barriers’ to access to prevention, vaccination and care for people (Manager, March 2022, WC). For example, people could decide where to test for COVID-19, had quick and easy access to vaccines, and to a wealth of information (provided via SMS, WhatsApp and call centres), received notifications of their test results via SMS with information on accessing support and next steps, and were encouraged to practice healthy behaviours (such as mask wearing, social distancing, cough etiquette, self-screening).

Participants shared how initiatives such as alternative medication distribution points, prepacked medication and multi-month dispensing (already widely available in the HIV programme) were adopted in the TB programme for the first time during COVID-19 to counteract some of the challenges to accessing healthcare patients were experiencing. For some TB patients, this involved limiting direct observation therapy (DOT) in the first 2 weeks of treatment, multi-month dispensing in the continuation phase, and home delivery of medication.

These innovations and differentiated models of care were considered important steps towards delivering TB care that would be more responsive to patient needs and preferences. However, participants also suggested that these innovations alone would not overcome some of the tensions in TB care, and what they believed was undermining the patient-centredness of the programme. Some managers referred to this as the ‘policing’ aspect of TB treatment. On the one hand, participants wanted to grant people taking TB treatment more responsibility for managing their care. On the other hand, they were concerned about the infectiousness of TB, non-adherence, loss to follow-up and poor health outcomes. This tension was most notable in the concerns and divergent opinions among our participants about whether TB patients should receive a positive TB test result via SMS, as with COVID-19 test results. Facility-level participants most often expressed concern about losing the opportunity to share a positive TB diagnosis with a patient and to provide information about the disease and treatment at the same time, as well as concerns about patient privacy. Manager participants were more often supportive of a shared responsibility for care and treatment on the part of the TB patient and the health service that could be facilitated by sending test results directly to clients and the health service.

### Community engagement

Manager participants in both provinces explained that involving community leaders and gatekeepers as allies in healthcare was crucial to the success of any health initiative. Such leaders and gatekeepers included those from faith-based organisations (e.g., church leaders), traditional leaders (e.g., kings and chiefs) and other community leaders. A manager explained:

In the past, the Department of Health … approached [health interventions] from a position of superiority … ‘we are the authority on this topic and we will tell you how to do it.’ …Through COVID, we’ve learnt that doesn’t work with communities, they need to be part of the process in terms of how you go about implementing new programmes…If these community leaders aren’t with you from the start…they become the biggest barrier at a community level because their influence and reach within the communities is so much and the trust that communities have in them is so much more than what they have in us (March 2022, WC).

In KZN, traditional healers were singled out as particularly important to the TB programme going forward as managers engaged them in the COVID-19 response. They understood that for many patients, traditional healers are a first healthcare contact. Participants believed that engaging with traditional healers as partners in health – providing training on the signs and symptoms of TB – was critical as they could serve as important referral mechanisms for patients to access care at health facilities.

Both managerial and patient-facing participants saw stark differences in public engagement for COVID-19 compared to that of TB. Whereas COVID-19 saw widespread public health campaigns driven from the national level, similar communication about TB was often focused on World TB Day, and the remainder of the year the onus fell on health workers to inform and educate communities. The sentiment among participants was that this communication was insufficient to engage communities and the public more broadly, and that,

…it is now up to the national level to prioritise TB education like they did with COVID. (Community Health Worker, April 2022, KZN).

### mHealth and telehealth technologies

The COVID-19 response spurred the creation of numerous mHealth technologies. These included the use of tablets/smartphones for COVID-19 contact tracing which allowed health workers to screen and document the information on site. Several patient information digital systems were also introduced, including a national COVID-19 screening app, public-facing COVID-19 data dashboards (interfaced with integrated public and private health information systems), SMS-based COVID-19 test result notifications sent directly to clients from the National Health Laboratory Service, and mass WhatsApp and SMS communication campaigns. Managers shared positive experiences of these apps and systems and believed there was potential to leverage and implement similar strategies for the TB programme. For example, one manager explained how digital apps allowed rapid reporting during the COVID-19 response, saying that ‘the information could flow …you could know what changed yesterday and your milestones’ (manager, March 2022, KZN). Another manager saw the potential of facilitating linkage to care with SMS-based TB test results. They explained that many patients visit urban areas for shopping and other recreation, test for TB, and go back home (to the rural areas) without receiving their result. With an SMS-based TB test result, the client could ‘get that SMS [with their positive test result] go to the closest facility, be able to follow the link [in the SMS] … to add your [test] results right there [at the health facility] and commence treatment’ (manager, March 2022, KZN).

The COVID-19 response also facilitated implementation of telehealth not explored at this scale in South Africa before. Managers in the WC, where a COVID-19 call centre was implemented for contact tracing and management and for providing general support to COVID-19 positive patients, told us how the call centre showed that ‘[we can] engage patients without them having to be in front of us’ (Manager, March 2022, WC). WC managers believed that there was potential to integrate the call centre with the Western Cape Provincial Health Data Centre (housed within the Western Cape Department of Health) to provide tailored telephonic support to TB patients.

### Rigorous contact tracing and management

A centralised contact tracing approach was followed for COVID-19 which involved lists of COVID-19 positive clients distributed to managers, with the responsibility to contact these clients telephonically and with home visits to offer information on isolation and care and to screen other contacts – a task usually delegated to counsellors and community health workers. Managers explained that for COVID-19 ‘they were even overdoing [contact tracing]’ (Manager, February 2022, KZN) and that ‘every single case and every single contact were either called or traced’ (Manager, February 2022, WC). Managers explained that while they followed a centralised process/system for COVID-19 contact tracing and management, TB ‘[contact tracing] is subject to the nuances of what is happening at facility-level and how functional or dysfunctional that system is’ (Manager, February 2022, WC). Significant resources were diverted from other programmes and made available for COVID-19 contact tracing, including human resources and transport, which enabled implementation.

### Widespread mask wearing

All participants lauded widespread mask wearing achieved in the COVID-19 response and supported continued mask wearing in health facilities for infection prevention control and to mitigate stigma. A manager explained:

…for the whole population to have gone through a phase of mask wearing is going to make mask wearing for prevention of transmission, including TB, much easier. (February 2022, WC).

## DISCUSSION

This study has identified eight facilitators of the COVID-19 response that could inform the TB programmatic response in South Africa. Participants emphasised the valuable lessons that can be drawn from these facilitators to guide the recovery and enhancement of the TB programme in the future. In particular, they highlighted the paramount significance of political will in driving increased accountability, ensuring access to necessary resources and fostering a sense of responsiveness and urgency in the implementation of the programme, similar to the approach adopted for COVID-19. They believed that there was limited political will in South Africa’s TB response and recognised it as key to improving TB service delivery and control efforts going forward. The transformation of South Africa’s HIV programme can similarly be attributed to the pivotal shift in political will that was catalysed by civil society movements. This shift played a vital role in achieving the crucial milestone of providing free access to antiretroviral treatment within the country’s public sector.^27^,^28^

The joint statement released by the WHO and The Civil Society Task Force on TB in 2022 calling for increased political will and accountability to ‘get progress to end TB back on track’^29^ echoes this sentiment. Limited political and donor will to address the global TB pandemic has been the persistent Achilles heel of TB control efforts worldwide.^18^ While South Africa has made notable progress to scale up a national TB response since implementing a national TB programme in 1994, historic inequalities, weak public health infrastructure, a growing HIV epidemic and rising rates of drug-resistant TB,^12^,^14^,^30^,^31^ mean that extraordinary commitment and effort from international donors, government and the civil society are needed. South Africa’s National Strategic Plan for HIV/AIDS, STIs, and TB 2017–2022 calls for an ‘all of government and all of society response’ to these diseases;^32^ however, this has failed to gain traction, with TB prevention and treatment efforts suffering poor policy implementation, limited resources and deprioritisation.^14^,^30^,^33^ In contrast, civil society endeavors to combat the TB epidemic at the community level have experienced limited success, especially when compared to the remarkable achievements seen in the context of the HIV epidemic. The success of HIV-related efforts is significantly grounded in the active involvement of people living with HIV in various aspects, including prevention, treatment, and care.^31^

There is evidence that the COVID-19 pandemic has brought renewed focus on TB globally and that in South Africa, lessons from the COVID-19 pandemic and response are being applied. In April 2021, the Western Cape Provincial government launched a multi-sectoral emergency response plan to reduce TB which included, among others, a public facing TB dashboard (as was available for COVID-19),^34^ TB awareness campaigns, targeted universal TB testing for high-risk groups, advocacy for psycho-social and nutrititional support interventions for patients, SMS notification of TB results, and improved diagnostics and focus on TB preventive therapy.^35^ The National Government has also committed to increasing TB screening and testing by releasing TB HealthCheck on WhatsApp to promote self-screening for TB, and acknowledging the innovations that have come from COVID-19 that should be leveraged for TB.^36^ Specifically, they recommend using mHealth technologies for screening, contact tracing, and adherence, incorporating home delivery of TB medications and easing access through inclusion in the Central Chronic Medicines Dispensing and Distribution (CCMDD) programme. These innovations have been shown to hold promise for supporting TB and other health programmes in the South African context.^37^,^38^ An initiative for targeted universal TB testing has also been nationally endorsed and its implementation supported by the President’s Emergency Plan for AIDS Relief (PEPFAR).^39^

There is considerable tension between how TB services are imagined and historically delivered and the potential to rethink and innovate TB services; TB services have seemed somewhat ‘stuck’ in the past.^40^ For example, many approaches and innovations implemented in the COVID-19 response, including multi-sectoral collaboration, contact tracing, mHealth technologies, integrated data systems and mask wearing, have met with limited success in the case of TB. Massive, decades-long investment into South Africa’s HIV and TB epidemic responses have enabled a comprehensive COVID-19 response.^12^ It is now crucial to apply the lessons learned from the COVID-19 response, armed with the understanding that these lessons can be effectively implemented through political commitment and determination. An easily implementable example is the practice of mask wearing. Prior to COVID-19, mask wearing as a prevention intervention was consistently met by low uptake by both health workers and people affected by TB; this could soon become another potentially missed opportunity for TB, as public acceptance declines alongside reduced COVID-19 numbers.^7^

The strengths of our study include the detailed qualitative interviews that allowed participants to reflect on their experiences and lessons and share them in an open-ended manner. The timing of the study is relevant in that the interviews were conducted 2 years into South Africa’s COVID-19 response, as national COVID-19 restrictions were being eased. This meant that participants had experienced the height of the COVID-19 response and were able to reflect on the response over time. Including two provinces in our study, each with different demographic and disease profiles allowed for diverse experiences to be captured. The similarity in lessons learnt from these two settings provide additional support for our findings. One of the primary limitations is the relatively small sample size, particularly in only two out of the nine provinces. Consequently, the transferability of these lessons to the national level may be limited.

## CONCLUSIONS

TB programmes in South Africa and globally have faced significant setbacks due to the impact of the COVID-19 pandemic and associated responses. However, our study reveals valuable insights from the COVID-19 response that can be applied to enhance TB programmatic management. The collaborations, technologies, and innovative health service delivery approaches inspired by COVID-19 present opportunities to maximise the benefits for the TB programme. Many of these approaches have already been endorsed and implemented within the South African health sector. Nevertheless, the true testament to a unified commitment to combat TB lies in the successful implementation of these strategies. To achieve national TB control goals, it is crucial to reinvest in the TB programme and prioritise it politically. Additionally, active engagement of civil society in both the short and long term is vital. These actions, coupled with political prioritisation and sustained support, will be instrumental in driving progress and achieving the desired outcomes in TB control efforts.
