# Supplementary material for: TB programme stakeholder views on lessons from the COVID-19 response in South Africa
Source: Public Health Action. 2023 Sep 21;13(3):97–103. doi: 10.5588/pha.23.0015 (PMC10446663; doi:10.5588/pha.23.0015)
Supplement: Supplementary file 1 [file iutld_pha_23.0015_supplementarydata1.pdf]

http://dx.doi.org/10.5588/pha.23.0015

TB programme stakeholder views on lessons from the COVID-19 response in South Africa

Supplementary Data A. Selected quotes from interviews that illustrate the eight facilitators of the speed and comprehensiveness of the COVID-19 response in South Africa, with codes from Table 1 represented.

**1. Political will**<br><br>Codes represented:<br>• *Challenges specific to TB*<br>• *Fear and uncertainty*<br>• *Integrated data systems*<br>• *Political will & commitment*<br>• *Contact management/tracing*<br>• *Multi-sectoral involvement*<br>• *Community engagement*<br>• *Key lesson*	'The urgency is because we had the Health Department and the political principals pulling in the same way. The Health Departments do what the policy makers tell it to do. If the Minister of Health says jump, you jump. In TB we don't get that political support for whatever we're trying to do. With COVID you didn't have so much civil society, kind of motivation – but you certainly had strong political commitment. You have to recognize that an emergency is different to your house is on fire. You respond differently than if you have a leaking roof. They are different contexts. But actually we did some things well, that we could replicate.' Manager, March 2022, WC<br><br>'TB has been neglected and as a TB programme we need to work hard to make TB visible, to make people more aware of TB. There's a culture where TB's only coming in the media on 24 March, because [it is World] TB Day. After that you don't hear nothing until the next year. It's not ok. Us in the TB programme, we should find a way of bringing TB every three months in the media so that we talk about it, and not just wait to speak about it once a year. In that way people will probably be more aware and will know about TB. We need to work more closely with some of our communication officers. I don't see TB being receiving attention that it deserves. COVID has exposed us on things we failed to do in the past. But there aren't only bad things with COVID. The positives are these lessons and how we did with COVID. A lot of these lessons can add a lot of value to TB, to HIV programmes, to other maternal and child health programmes. We just need to take these serious and begin to improve our programmes.' Manager, March 2022, WC and KZN<br><br>'It's not just a matter of resources. I found over the years of working with TB that you get those people that are interested in TB and those that are not. The ones that are interested in TB tends to perform better, have more focus and have better outcomes at their facilities. And those that are not really concerned so much about TB, and don't really see the importance of what we do, you'll find that those other facilities that actually struggle and don't perform well [with caring for their] patients. A lot needs to be done to ensure that resources are available for the TB programme, but also a lot has to be done with people and changing their [frontline health workers'] minds so they can understand how they can actually impact the programme. TB often has to beg for resources, whereas if you look at the HIV programme, resources just come flowing from all directions. NGOs that come, they want to work with HIV, a government wants to give funding for HIV, and TB just gets the scraps that fall off, or things that we try and manipulate into the HIV programme, you know?' Manager, March 2022, KZN<br><br>'Just as we had all this hype created around COVID-19 we should also get the same hype for TB. Have more broadcastings, more of your media, speaking about TB…Have you seen any social media? Adverts about TB? I personally haven't seen anything on TV, on TV or radio yet, about TB. Everybody is still ranting and raving about COVID and the need to vaccinate… So why can't we get the same hype about TB. I mean it's TB month, we should have a lot of information flying around on social media, newspapers, and so forth, to educate people about TB, that people understand what TB is. Because a lot of times when you interview patients you will find that the understanding of TB is very poor, and this is the reason for the reluctance to seek care, the fear. People still think that you have TB [you] will be taken away and sent to an isolation facility, and only see your family after six months. You know how it used [to be in] the 80s? So, there's a lot of fear around it and I think we should be talking more, just as much as talking about COVID. We need to get people to understand what TB is, how its treated, how you can keep those around you safe, if you are diagnosed with TB, and the importance of early treatment.' Manager, March 2022, KZN<br><br>'They're trying to apply some of those learnings [from the COVID WOZA (Whole of Society Approach approach)] also to TB so that you've got the Premier's [TB] response plan where he does advocate for a whole of society approach. So that's where the province would like to be going [with TB]. But in practice you don't see it really taking off, you know. So, there's a lot more work to be done on that, and I think

	that's what province wants to do, it wants to include everybody and to have that Department of Education, and across the board people being aware of the TB response plan and be increasing screening, increasing diagnosis, because of that awareness. A way forward would be to try and apply those COVID learnings with the case and contact tracing and then to apply the Premier's high-level plan and to actually get that to infiltrate at an operational level, and actually to have some yield and some uptake.' Manager, February 2022, WC<br><br>'I don't know how they actually integrated the [public and private] systems, but you know when they reported the COVID cases, they would report what was happening also at the private sector. So, I haven't seen any good private public partnership as we had during COVID. So, I think all the systems were also actually…interfaced. This was a few months into COVID. This is what actually taught us that things can actually move fast. You thought, can people move this fast? If you had asked me before COVID, I was like, no things never went this fast.' Manager, March 2022, KZN<br><br>'The other thing I just really also want to mention [that was important in the COVID-19 response] is that your political buy in and support in the response, you know, from the president, to the premier, to senior managers within the Department of Health getting out there, being visible, speaking about this. And even community leaders, you know, so faith-based sector, influencers on social media and so forth. And so, I think that is something that we need to think about, you know, how do we kind of generate that same energy and effort for the TB response?' Manager, March 2022, WC<br><br>'For us in the Western Cape [province], COVID has forced our political leaders to be much more active in the health space, and to appreciate the impact of not addressing some of the serious health priorities, you know, what is the possible health impact that that can have on society at large. So, there's a much greater understanding amongst high level political leadership of how crucial the health system is within the bigger societal system. And for us in the Western Cape we've got a TB dashboard, yay! And if there wasn't a COVID [dash]board, we would never have gotten that. Same with the [TB Check self-screening] app. So, there are definitely some small wins that we can take out of this.' Manager, March 2022, WC
**2. Rapid policy development**<br><br>Codes represented:<br>• *Challenges specific to TB*<br>• *Multi-sectoral involvement*<br>• *Rapid policy development*<br>• *Political will & commitment*<br>• *Key lesson*	'I think prior to COVID, everything was just taking long and by the way, other things still take very, very long. But COVID shows that we can respond if we want, you know, if we want to bring in leadership, if we want to do a good job, we can respond. We can respond fast. And the COVID response has been fast, and I don't see anything wrong with it. The evidence is good. Most things for me are just in order. And fast. In the olden days people had their perception that things must take years, then they mature in the drawers, and they come out with quality. I don't believe in that…My sense is that in the past, if you have also been complaining about the slow pace, you are now one of those people who have been very frustrated in government because of the speed that we do things, and I was always surprised that people were surprised that I am unhappy about the speed. Which means, most people were thinking that it's just ok. Wait for this, wait for that. They wouldn't even ask what they are waiting for. Just waiting…Now somebody wants to make a big announcement during the World TB Day. So you wait until it's World TB Day, whether it takes you nine months to reach that TB Day or six months, it doesn't matter. This is not okay. You know. I don't like it, but I think people are now awake. More people now are complaining about the speed. They are able to compare, because they see that we are doing certain things fast as government now they're comparing, and they're starting to talk, and I think that's probably what is happening now.' Manager, March 2022, WC and KZN<br><br>'During the COVID pandemic policies were passed overnight. Literally. Yet, if you want a TB policy to be passed, it takes like, you know, 6 to 18 months. So, I'm trying to get people to understand that actually by being more responsive you sometimes have a better impact than by having the perfect answer.' Manager, March 2022, Manager, WC<br><br>'We had a fabulous team that was actually drafting the policies. We saw more COVID policies of, I think higher quality coming out of the provincial office in the space of a month, than we saw coming out over years before in all the programme areas. And it was one of the learnings, you know, we had various learning sessions along the way and in one of those we actually reflected back and said if we could just learn what really enabled that policy process and help it apply to other programme policy areas that would really help. Because from my field, from HAST (HIV, AIDS, STI's and TB) we've really been struggling with some of the policies that

	needed to have emerged but haven't… I think what happened is there was an unprecedented collaboration and involvement of public health specialists in the policy process, which before had been in programme specific silos. And I think it just brought a whole other level of quality to it. And then I think with the urgency. I mean, they [the Western Cape Government] did then deploy people to do that, you know dedicated [people]. And then with COVID there was engagement with public health specialists, both employed by the Department [of Health], but also that gave support from their academic institutions in a way that I haven't seen, also to a degree, that I haven't seen happen [before].' Manager, February 2022, WC
**3. Multi-sectoral collaboration**<br><br>Codes represented:<br>• *Challenges specific to TB*<br>• *Multi-sectoral involvement*<br>• *Rapid policy development*<br>• *Integrated data systems*<br>• *Political will & commitment*<br>• *Community engagement*<br>• *Key lesson*	'There was so much expertise, so when people work together, it really was amazing. Another lesson we could learn for TB. From public health specialists to professors and radiologists, everyone came on board. It was just amazing, so you wonder why not for TB?' Manager, February 2022, WC<br><br>'Partners came on board, the businesses, private sector, other government departments, they were pitching in… people were willing to develop posters to get the communications out there, whether it was via social media or traditional methods of communication in terms of leaflets, and posters, and billboards, radio ads and TV ads. So there was a lot more buy-in from outside the health sector to make these sorts of COVID communications… That is something that we would like to have in the [TB] response, but it would take a bit of work to convince people that the impact of TB is on par with COVID.' Manager, March 2022, WC<br><br>'It is around those partnerships. You know, how businesses really opened their doors. And even thinking of COVID-19 vaccination, for example. Workplaces have opened their doors and said, you know, come in and they've given staff the time off to get vaccinated and so forth. And we really don't see that for TB. We don't see people being given time off to go to facilities to collect the sputums. We don't see workplaces say "no, come and do HIV testing and TB screening at our workplace today." So it's just those types of… the partnerships with the private sector [are] key, the resources that are made available through other avenues that's outside of the health sector, for the COVID response.' Manager, March 2022, WC<br><br>'What we can learn from COVID for TB starts with collaboration between private sector and public sector. Every evening, every day when DOH (Department of Health) sends results of people with COVID, those results come from both public and private. Something we failed to do for TB all these years. We need to have a system whereby we get data from private and public together to inform the nation. Also, working together in terms of exchanging notes with regards to [TB] management …we don't know how many are in the private sector of South Africa. We don't know how they are managed. What are their treatment regimens.' Manager, March 2022, WC and KZN<br><br>'When you have your funders and implementers and everyone in one room…you agree on something now, somebody comes and says, "ok, I will try that out". And then by the time they report [that] this can work, the funder is lining up to see how much this would cost, and then …fund it… That is what made the whole COVID thing to rapidly move …most of the strategies that we proposed… [Usually] the support partner comes with already negotiated activities, even when you say "no", as a province, "this is what I need" …the needs-analysis will already [have] been done."' Manager, March 2022, KZN<br><br>'COVID was an effective response. The way we responded as a district, it showed that it was effective, I would say. Everybody was part and parcel of the COVID [response]. It's the response from management, the buy-in from management. The way people – they were responsive to COVID. We wish they could respond the same to TB…The directive from province to assemble and activate teams to mount a COVID response was instrumental to this mobilising, and there were many more teams available [for the COVID-19 response] than there are for other programmes.' Manager, February 2022, KZN
**4. mHealth and telehealth technologies**	'I think most of all [what improved] is the reporting because they had digital apps [for COVID-19] and the information could flow…you could know what changed yesterday and kind of get your kind of milestones. You would know, ok the index case had 20 contacts. But you could check in the system you know, so far, they've scanned only five. Whereas the contact management for TB is still paper based, it is still a long way to get there.' Manager, March 2022, KZN

Codes represented:<br>• *Contact management/tracing*<br>• *Screening/testing*<br>• *mHealth technologies*<br>• *Challenges specific to TB*<br>• *Challenges specific to people affected by TB*<br>• *Patient-centred care*<br>• *Integrated data systems*<br>• *Telehealth/call centre*<br>• *Political will & commitment*<br>• *Community engagement*<br>• *Key lesson*	'What I …thought that we could actually use in the TB programme was their ability to actually screen and document the information on site [in the COVID-19 response]. You know, like with a lot of these teams, they went out with tablets for the COVID-19, when they found the patient it was entered into the tablet, along with the contacts, and all the contacts were found, they were screened and tested, at place of residence. So for me that was an excellent idea to actually ensure that, because your contacts were populated onto the computer system or tablet, and as you as they got to them, this contact was found, they were screened and tested, you know.' Manager, March 2022, KZN<br><br>'The…results being SMS'd to you, I think that was brilliant. That's something that would work very well with patients, especially TB, because one of our challenges in the urban areas is that you have people that come in through your urban settings to do shopping, restaurants, whatever the case, from the rural areas. And they come in here [in the urban centre] to get tested [for TB] here and then go back. And when it comes time to find them, it becomes a challenge because a lot of them give you incorrect addresses because of fear. However if we had a system like we had for COVID where they get an SMS…and following that link from that SMS, you can actually get the result, and the patient could test in central [town], and then go out to one of the rural areas, and when they get that SMS go to the closest facility, be able to follow the link and be able to add your results right there and commence treatment with you. That, would be awesome.' Manager, March 2022, KZN<br><br>'[The COVID-19 screening app] is very user friendly. It has your questions for COVID screening. I think asking those questions might take, maybe two, three minutes to actually fill that on the app. And then it classifies your patients as someone who needs to be tested, and you also record that you are testing the patients. And it has a prompter. It will also call back the result, then patients get their results via SMS. By the time the health worker knows, the patient also knows. And what is also nice, even the contact management, because some of the patients were diagnosed through the private sector, but the public sector could also get the information from the private sector. I don't know how they actually integrated the systems, but you know when they reported the COVID cases, they would report what was happening also at the private sector. So I haven't seen any good private public partnership as we had during COVID. So I think all the systems were also actually… interfaced. This was a few months into COVID. This is what actually taught us that things can actually move fast. You thought, can people move this fast? If you had asked me before COVID, I was like, no things never went this fast.' Manager, March 2022, KZN<br><br>'The way COVID information has been distributed is more than what has been done for TB. I feel like COVID is of great importance and TB is side-lined. COVID has WhatsApp, TB doesn't. Even on Facebook, if I get on Facebook, I come across COVID surveys but I have never come across such for TB. Using similar strategies for TB will help in increasing awareness about TB. If TB patients can have an app, or an SMS line to remind them of their clinic visit for treatment collection.' TB nurse, May 2022, KZN<br><br>'You would even get [your COVID-19 test result within] 24 hours, you would get a result…That's the thing about digital, using technology. We must go the digital way. Because our [lost to follow-up], both initial patients that come to the clinic, make the effort, are sick, take the sputum, don't come for treatment. That's a big, big gap, we need to get those patients on treatment, because they are obviously infectious, they're at risk, they may die, they may transmit, and so to get that. So everyone, like the NHLS sends the SMS or your private labs, to say you test positive. But the call centre must be used [to] start phoning newly TB diagnosed patients, or patients who were diagnosed but haven't pitched up at the clinic, say positive, please report at the clinic. And if you're feeling sick, we link them to a clinician like we did with COVID. All that can be done. NHLS sending results as well, positive sputum results to patients. There's been some resistance about confidentiality and stuff I think with HIV, it's a bit different. But I mean we did it for COVID, an infectious disease, why can't we do it for TB? I think, those two things, that SMS notification and the call centre will work brilliantly for TB. It will be a minority who doesn't like to be called. Especially private people. But I don't think by and large the majority felt so nice that someone actually called them to check on them, you know, how are you doing, how can I help you?' Manager, February 2022, WC

	‘So the patient profile that we had with the COVID is a little bit different from what we have with our TB patients. A lot of the [COVID] patients that we phoned were actually medical aid patients or private patients, younger patients and they were not very sick in most cases. So, you could have a conversation with them and talk to them about their contacts and everything and it was not really anything challenging in that kind of a conversation. Whereas with TB patients they tend to be much sicker. From my clinical experience a lot of them are much sicker. And then there's complications with their comorbidities and HIV and other things that they might have. And then also, you have to take some time to counsel them on their clinical condition before you get to talking about contacts and other things. So, it's a bit different. With COVID it was fairly straightforward in most cases. There were some that were bit complicated, but most cases were quite straightforward…Some of [the approach can be replicated]. We are trying to implement some of these learnings with using the COVID call centre at the moment, the concept of phoning the person who has the disease and then asking about contacts may be applicable. And so we're going to try it out and see how it goes, taking into account that it's a different kind of a patient profile… [Another thing is that the call centre] people are not medically trained. So, they have a set script where they ask particular questions and then they can refer [patients] to somebody in the district if they need to be followed up, or they can refer to a clinician. So using that kind of a model for TB is the idea that we were trying to sort of pilot now. Where you phoned somebody who's diagnosed with TB and then check how they are, and then check on who their contacts are, see if there’s anyone symptomatic and then link them to your community-based services and facility-based services, and maybe a clinician if they are quite sick. Because I mean, just like COVID there was a possibility of the person’s condition deteriorating quite quickly in the first two weeks. That does happen with TB patients as well. So that's one of the things that we'll be looking at here as well. Just checking if the patient is clinically stable, if they have any warning signs and that kind of thing.’ Manager, February 2022, WC<br><br>‘With COVID, for some reason it just became very easy to just send people SMS’s. That was a surprising thing, you know, because suddenly everybody had a phone. And previously our experience clinically was, if you phone people, you could never get hold of them, especially in public facilities. You always have the wrong phone number, or it doesn't work, like goes to the wrong person. But all of a sudden, with COVID it was such a successful thing. So that's the other part of the project that we'd want to coordinate is we need to also support it with the process of capturing correct details of people. Asking people, are you sure? What's your phone number and is it working and functional and all of that stuff. Because I think that that might be an issue in the public sector. There's a high turnover of phone numbers. Phones get lost, phones get broken, and all of that stuff. Whereas [in the] private sector [people] tend to have the same number and phone for quite some time.’ Manager, February 2022, WC<br><br>‘There was also various other initiatives that evolved out of [the call centre]. So the one which we are particularly learning from now to help inform HIV and TB, was to pick up high risk individuals for COVID-19 death who were uncontrolled diabetics. So again, using the data coming out of the PHDC [provincial health data centre], it could match who was being diagnosed with COVID-19 and who was known in the system to be diabetic. And so those people could be contacted to have risk profiling done to pick them up early and make sure they were referred into care. Because we have this fabulous resource in the provincial health data centre, which brings together a whole lot of different sources of data. So that’s why I think is also one of the reasons the Western Cape government was able to leverage on that and very quickly use it to apply to COVID and bring the COVID data onto that platform to do these kinds of analysis and perform work processes. But pre COVID, we'd started work and then we had grant staff employed literally as COVID started I think it was March 2020 – to try and use the data under the PHDC for at risk, and especially LTFU HIV and TB patients. And now we're saying well, how do we add one of these telehealth aspects that was tried and tested and had its application during COVID with the diabetic project. So we we're trying to see which aspects of it to apply [to HIV and TB].’ Manager, February 2022, WC<br><br>‘The call centre that was set up for COVID, people really seem to enjoy that. Having somebody who's available to answer your question so you don't have to go and see a clinician you can actually just pick up a phone and have your question responded to. Obviously if it's something complicated, you may end up needing to go and see a clinician, but it's not for every single query…So the province is gonna start testing the call centre for linkage for TB treatment, and then maybe also for retention on TB and ART, but they are starting with testing a call centre approach to linkage.’ Manager, March 2022, WC

	'We realized in COVID that we can engage patients without them having to be in front of us. And I think that that's something that can be brought up.' Manager, March 2022, WC<br><br>'We only used phone calls to contact TB patients; I have little knowledge about mHealth technologies, but I feel they can be useful on the TB programmes. As this is a rural area, the health facilities are far from households. These technologies will help the people that are far from the facilities to be able to screen themselves and access the help they need.' Community Health Worker, April 2022, KZN
**5. Rigorous contact tracing**<br><br>Codes represented:<br>• *COVID-19 impact on TB service/programme*<br>• *Contact management/tracing*<br>• *mHealth technologies*<br>• *Multi-sectoral involvement*<br>• *Integrated data systems*<br>• *Telehealth/call centre*<br>• *Political will & commitment*<br>• *Poor TB programme implementation*<br>• *Key lesson*	'Contact tracing with COVID, it was done properly. We saw teams going out and actually doing it. With TB it was never done consistently, although it always appeared in all the guidelines…You always get the report in many places that there's no transport and this and that, but suddenly for COVID there was transport. So I think we must just be effective. The things that were done very well with COVID was supposed to be done with TB, but they were never done. So if we can really make sure through our provincial politicians, you know, the bosses, that we get the resources to do this for TB you know it's going to be very helpful.' Manager, March 2022, WC and KZN<br><br>'It's the focus that actually made so much of a difference with regards to the COVID being so successful in the tracing and testing of patients. You find that because the entire district was watching, everybody had to do what they needed to do. And I think when the focus falls away, then people also tend to be a little more relaxed and not move so swiftly to get to these patients. It is the pressure, but also, I think monitoring is very important. You'll find that we have to, we have a similar situation in our district as well where you have a Primary Health care manager whose focused and she monitors her teams closely as well as operational managers who monitor the [tracing] teams closely. We have a much better response and much better outcomes in those sub-districts and facilities. Where you have less focus and less supervision and monitoring, you find that it doesn't work.' Manager, March 2022, KZN<br><br>'They were even overdoing [contact tracing with COVID-19]. Because they could not wait for a few days to do the tracing, they would just go to the family where the index [COVID-19] case has been identified for screening and contact tracing, weekends, during the day. I think if we could copy that process of doing our contact tracing, we would really win TB. When there is an index patient that is positive [for TB], we must say, "we need to go, there is an outbreak." One positive case of TB is an outbreak. So that we can go and we make sure we try to eliminate the transmission of TB.' Manager, February 2022, KZN<br><br>'For COVID, every single case and every single contact were either called or traced. If we couldn't find them, we'd send someone to do a home visit. And this was done well, through the [COVID-19] contact centre, but also through [our tracer] teams. For TB it's done very poorly. They just make a list in the TB folder and you're not sure if anyone's been assessed or even a home visit done. To see that it can be done if you do it the proper way and with the resources. The learning is it can be done.' Manager, March 2022, WC<br><br>The participant explained that the Western Cape Government was recruiting from health programmes to support the COVID-19 response, and the participant was asked to join as a case manager. They explained that as the case load became bigger they started recruiting students from the Medical Faculties to help with case and contact tracing. These case and contact tracing teams were called 'pods', and they supervised one of the big pods: "the Provincial Health Data Centre would give us line lists of the newly diagnosed people with COVID and their contact details would be on there as well, because PHDC (the Provincial Health Data Centre) was getting daily feeds from the labs – private and NHLS (National Health Laboratory Service), so we would get line lists and in the morning those line lists would be delegated to different pods. Some of the patients needed clinical support, so then I would be the contact person to phone them back and just check how they were doing and see what the emergency was. But most of the time we just had a template that you had to populate with the patient details and with their contacts and then you would also just check on whether the contacts was symptomatic or not, and you would give them advice, you know, staying at home for the 14 days and those sort of things. What to do if you get short of breath and that sort of thing. Because at that stage it [COVID] was still very new. So a lot of people were quite panicked about what was going on. In fact, with us starting right at the beginning when the

	first few patients started dying, that was like a really big development for us, you know, seeing this evolve. There was lot of fear and anxiety about what was going to happen in the province… Eventually the programme became so big that we had to recruit the 6th year [medical] students to the pods for additional support. University classes had been suspended at the time as universities were closed, and so this was a good solution to bring students into the COVID response as part of their 6th year rotation, while also being able to give them relevant experience.' Manager, February 2022, WC<br><br>'A lot of us [in the Department of Health] just got pulled in and instructed that, you know, we need to be part of this contact tracing team because we didn't have any other human resource capacity that could do this. So firstly, we've never seen something like that happen for TB to say "here are these lists of TB contacts… here's a phone, you're going to phone them now." So that sense of urgency and priority definitely hasn't been there for TB. We know TB contact tracing is decentralized all the way down to the facility level, to do follow up and contact tracing. But I think that is something through the contact centre that we're now learning, that we can do this for our TB clients, you know, if we extract the data from our Provincial Health Data Centre and we have contact details, we actually can start following up with TB contacts. We can start following up with clients who've missed their ARV [antiretroviral] pickup dates, even our TB lost to follow-up clients, not just contact tracing.' Manager, March 2022, WC<br><br>'[Contact management teams] came from all stakeholders from [government] so each area had their own teams that managed cases, so we had now to distribute that to our case managers. And we included our Environmental Health [department], they focused more on the contacts of the patients. It was a massive programme… At the beginning we used to call people daily, we used to work on weekends, to get cases on weekends, Saturday night, Sunday night… But it was the province, it was City, academics, it was NPOs (non-profit organisations), it was…whatever cadre of staff at the hospital, everyone got involved, even public health specialists at UCT (the University of Cape Town), [University of] Stellenbosch, UWC [University of the Western Cape]. They actually all phoned patients, so it was a multisectoral response…everyone across the board. It doesn't matter what your level or your rank, or your position. Everyone did it in the beginning… It was important, because if we didn't call them, what if that patient needed oxygen, needed to be hospitalised, so especially that initial call to make sure you're ok, no he's stable, do this do that. So that put a lot of pressure on our teams at facility level.' Manager, February 2022, WC
**6. Patient-centred models of care delivery**<br><br>Codes represented:<br>• *Actions to support TB patients/service during COVID-19*<br>• *Challenges specific to TB*<br>• *Patient-centred care*<br>• *Political will & commitment*<br>• *TB stigma*<br>• *Community engagement*<br>• *Key lesson*	'Even pre-COVID, I always thought, do you really need someone coming to the clinic every day you know? It's almost, like the policing thing. It's a bit... We should change this whole approach, and it should be more supportive. And put a bit more trust in patients and their families, and treatment supporting at home. Rather than asking someone to come daily to the clinic, to watch them swallow a tablet. Which doesn't get done if you ask me. If you're taking MDR (multi-drug resistant TB) treatment you're taking 20 tablets, and you're a nurse, and you got 10 patients, there's no way you're going to watch them swallowing 20 tablets at your clinic. So, DOTs (Directly Observed Therapy) is outdated, it doesn't work. Because in spite of all that [the surveillance] we still have a problem with TB, and high LTFU (loss to follow-up). So different models [are needed], giving the patient more responsibility, longer amounts of treatment, more community-based treatment, even if you send people to patients' homes, to go check on adherence, you know, community health workers. There's lots of models you can use, you don't have to bring people to clinic. Unless it's absolutely necessary for their blood or clinic check-up.' Manager, February 2022, WC<br><br>'We are now trying to get TB [test] results sent directly to patients… We've finally managed to get them [Western Cape Department of Health] to agree to send a negative result to a patient. But for positives, they only agreed to send a message to say they need to come to the clinic. So [there's] a lot of exceptionalism around TB because we're so worried about it, but … we could put information in the hands of the patient. Self-interest is the best way to get something acted upon. You know if someone sends you a result based on your test result, [as a patient you are] much more likely to take action [on a positive test result] than a nurse who has received 30 test results. So the health system can still have backup measures, but at least put the decisions in the hands of the patients and then a plurality of options. So, you know, even if we think about TB testing, for example, sputum collection. You know, why do you have a stand in a queue for half a day to provide sputum if you've got a cough? Can't we do that more efficiently? With COVID we had all these multiple points where you could go and get a test…You waited 10 minutes [or half an hour] and then you

	got your test... And similarly with treatment I think we're starting to recognize that if we lower the barriers [to access services, people will come], people don't want to go and queue in facilities. It's understandable…There's a whole lot of reasons [why they struggle to access services], you know, economically, family responsibilities, and just the frustration of these dysfunctional overworked systems.' Manager, March 2022, WC<br><br>'[For TB] there were national protocols that were suggesting delivering TB medication at home or through a so-called alternative pick up points [during COVID-19]. Our opinion was that it would be the part of the [TB] service we would rather protect at facilities. We thought it was too risky to try and put [TB medication] into a system that we hadn't already trialled and were confident about. ART patients only qualify for that kind of chronic medication offsite dispensing once they are stable. That form of interface is without clinician oversight at the time. So, TB patients, they don't as quickly get into that [stable] space if at all, before it's actually the end of the [treatment] journey. And [they need to come to the clinic] for the follow up tests which are needed in the TB programme month two and five, so it doesn't give you much space to…decant people into an alternative system.' Manager, February 2022, WC<br><br>'One thing we said [about the COVID-19 response], whatever learnings we got there, why should we stop. So, we've continued these good practices and we're trying to even see how we can expand you know, to do more of these home [treatment] deliveries, or multi month dispensing, even for prevention. There are some good models in [a large urban township], where they started screening patients at home, and contacts at home, and putting them on prevention at home. Not even coming to the clinic here, for TB… [These innovations are financially feasible] if you have support, if you have funding, donor funding, a lot of the NPOs (non-profit organisations] have resources. And the patients love it, and the thing is, what about the poor patient? What about the cost for them to come to the clinic every day? And the food, and the days off work? You must look at that cost. Cost of that versus the cost to the health system. So, you must look at [that]. Everything we do must be to suit the patients, not the other way around.' Manager, February 2022, WC<br><br>'By and the large the majority of my patients, they want to come [to the clinic], they want to finish their treatment. But some of that [is about] the way you support them, you know, not shouting at people when they come back after defaulting, you know, be open and say, "We missed you, what was the reasons?" Asking open ended question rather than being confrontational. So that's a big thing, with the staff attitudes and the "Welcome back [campaign]." The "Welcome back campaign," we start to tell people, you know, when patients come back, welcome them. Put them in the appointment system. They've actually come back, so be happy that they returned, you know. So that's how I practice, and my patients have very good outcomes. My LTFU (loss to follow-up) in my cohort of patients is low. And I teach the nurses the same set of principles and ethos. The way you care for patients and get other patients that have been cured before as peer supports, you know. That's the way to go. Listening to doctors and nurses is sometimes [difficult] you know, listening to someone who has gone through the experience, the journey, is better.' Manager, February 2022, WC<br><br>'Most of the participants that are initiated on TB treatment are from the community, it is a duty of a [community health worker] to make sure that these people adhere to treatment. For the people in my area, I make it my responsibility to visit them in their homes to see if they are taking the medication accordingly and to assess if they are having any challenges on their treatment journey and I remind them of their next clinic visit dates. You find that some patients are very sick, and they forget about their clinic dates, that is where we intervene and remind them of the treatment process.' Community Health Worker, April 2022, KZN
**7. Community engagement**<br><br>Codes represented:	'COVID awareness was of great paramount compared to TB. It did happen that you find information about TB on social media but for COVID it was done to a greater extent. COVID information was spread from the national level but for TB it is only the health care workers at the ground level from the clinics. We do enough on the ground to educate about TB, it is now up to the national level to prioritise TB education like they did with COVID.' Community Health Worker, April 2022, KZN<br><br>'The community people…are not taking TB seriously. So, what we did some time last year … I organised a health talk with the traditional healers for instance and also the traditional leaders at the community level, the faith-based organizations, because those

• *Actions to support TB patients/service during COVID-19*<br>• *Challenges specific to TB*<br>• *Community concern about TB*<br>• *Fear and uncertainty*<br>• *Multi-sectoral involvement*<br>• *Routine data publicly available*<br>• *Political will & commitment*<br>• *TB stigma*<br>• *Community engagement*<br>• *Key lesson*	are the influential people in the community that could influence these people to come into the facility and be investigated for TB. We had a really, really good health talk that took about three days…So yeah, basically if we target those influential people that will be able to influence our community to actually seek for help, not go to traditional healers when they see that they have got the signs and symptoms of TB.' Manager, May 2022, KZN<br><br>'We need to think about …how do we kind of generate that same energy and effort for the TB response [as we did with COVID-19]… Your faith-based sector, your church leaders, your community leaders, you know, we had the auntie running the soup kitchen promoting vaccinations for us, it's more around how do we empower people to take the message forward? Of course, you need a little bit of resources upfront, you know, to do that. But the impact is kind of long-term. I think we've learnt some of the ingredients for the recipe in terms of engagement. So that is also something at a higher level in the Department [of Health] that has been quite a key lesson for us, in terms of community engagement, stakeholder engagement, and how you take those stakeholders on a journey with you. In the past the Department of Health has really approached [health interventions] from a position of superiority, you know, "we are the authority on this topic, and we will tell you how to do it." And through COVID we've learnt that that doesn't work with communities, they need to be part of the process in terms of how you go about implementing new programmes… If these community leaders aren't with you from the start, and you walk a journey together, they become the biggest barrier at a community level because their influence and reach within the communities is so much and the trust that communities have in them is so much more than what they have in us.' Manager, March 2022, WC<br><br>'Symptoms of COVID or being diagnosed with COVID is not associated with any other sickness whereas if you are diagnosed with TB people automatically assume that you have HIV. People do not know that it is possible to have TB but not have HIV. I wish people can be open to their peers about their sickness so that people will see how these sicknesses are related and how they are not related. If we can also have community outreach teams that educate about such health issues even after COVID. Community health education should be an ongoing thing.' TB nurse, May 2022, KZN<br><br>'What we can take [from the COVID-19 response] is the way information about COVID was disseminated. The only thing that needs improvement in the TB department is education. People do not fear TB, they fear COVID the most. They fear COVID because it has killed a lot of people in such a short period of time. TB has been with us for the longest time it has become normal. People are not well educated about the similarities between these two viruses as they both affect the way of breathing.' TB nurse, May 2022, KZN<br><br>'There have been so many awarenesses (*sic*) and everything and the community health teams are in the communities giving health education and so on [about COVID-19]. But we still having this problem of TB. I think the main thing if we target the influential people at the community level that is where we could be able to tackle these things and if we target the traditional health healers in the community, that is where we've been. Because honestly, if our patients at the current level they are sick or they feel sick, the first point or the first people that they will consult is traditional healers before they could go into the houses. And so, if we target those people we teach them so that they teach the community about the TB programme and so on. I think we will have a great turn up at the facilities, health facilities that we have, and also teach those people to identify the signs and symptoms of TB. That this patient may be having TB, so I can refer the patient to the clinic to be investigated for TB. So, I believe so because they listen to these influential people at the community level. They listen to their pastors, they listen to their kings, and also the community leaders and also, they listen to their traditional healers as well.' Manager, May 2022, KZN<br><br>'I also think if maybe for the [TB] programme. I know this media awareness campaigns are actually quite expensive, radio airtime is expensive, but it will [have an impact]. You know because people do die more of TB than other diseases. So, maybe making people aware, and broadcast the numbers of people that still get TB in this day and age. Something similar to what was happening with COVID. Although even with COVID you know, there are also connotations, you need to also kind of review your strategies, your communication strategies all the time because people have gotten used to whatever information you give them. They're no longer

	as scared as they were of COVID, as they were before. And the nine strategies to actually prevent COVID, people have really, you know, the wearing of masks, the social distancing, those have toned a bit. So, I guess, the keeping up of reminding people is also important.' Manager, March 2022, KZN<br><br>'It would actually benefit the community as well having the [TB] data out there because they will see the seriousness of the disease, of how the disease is quickly spreading. Same as for COVID, the data was out there. They could see that this is spreading quickly, so they need to act swiftly. So, if we're doing that also for TB…people will be able to able to act up swiftly they will be able to go into the facilities to be investigated for TB and so on. Because currently we are struggling in getting people to be investigated, they are always denying signs and so on. So yeah, if we get in the data out there, it will actually work.' Manager, May 2022, KZN
**8. Widespread mask wearing**<br><br>Codes represented:<br>• *Mask wearing*<br>• *Challenges specific to TB*<br>• *Political will & commitment*<br>• *Poor TB programme implementation*<br>• *TB stigma*<br>• *Community engagement*<br>• *Key lesson*	'[Mask wearing] is one plus for the TB programme. We struggle at our facilities [to get people to wear masks], we always get everyone who are waiting, give them a paper mask. So, everyone had a mask, you know, so not just the clients of TB. So, I think now that's becoming a bit more acceptable in the communities, in the clinics, so that's positive. So, it's [mask wearing] become like an accepted thing. So that stigma, there's still a massive stigma for TB and DR-TB (drug-resistant TB) but I think it's helped a lot, mask wearing. It's definitely more accepted now. You will stand out more if you don't wear a mask.' Manager, February 2022, WC<br><br>'I think the mask wearing has played a significant role [in prevention] because we've seen a decline in new positive TB cases, so the mask has definitely played a role. We've been trying to get people to wear a mask if they are sitting at a facility for an eternity I think (laughs). We've always been advocating for nurses to identify complications and for people to wear surgical masks. And it's been a nightmare to get off the ground, people lose their masks. And thanks to COVID we now have everyone wearing masks, including nurses, 'cause we've also had a struggle to get health care professionals, not just nurses [to wear masks]. I'm talking about doctors and everybody else. People refuse to wear masks. You would only find a TB nurse would put on a mask. Nobody else. Thanks to COVID everybody's with a mask, and I think that has played a significant role in helping with the spread of TB.' Manager, March 2022, KZN<br><br>'The masks have become a norm now. Everybody has to wear a mask, so it's not like…people think you're crazy: 'Why are you wearing a mask in public, what is wrong with you?' They automatically think you are sick, maybe you're a cancer patient, you have TB, whatever the reason. Or they just think you're a germaphobe. But TB/HIV there is still stigma attached to that. … some of the times these medications I have to decant in a clear bag, with no information on there, because they don't want their family people or whoever knowing about it. They'll be so discreet about it. I have clients who don't even want to go to the pharmacy to get their medication because they see somebody that they know here.' TB nurse, February 2022, WC<br><br>'The mask wearing it doesn't have a stigma per se, but my opinion or my view is that if we could continue wearing masks, because we are used to them in any case, they protect us against the COVID, they protect us, protect other people against TB as well. So, if you …feel like you've got fluish symptoms, you've got TB symptoms, then it is advisable that everyone should wear a mask. We're always doing that. But at the facility level where we could identify any person that has a cough in the waiting area, then that person would be given a mask to wear. So, if we continue with that with that narrative, I believe we could be able to prevent so much.' Manager, June 2022, KZN<br><br>'The fact that we could mandate [mask wearing], and it would be great if that can continue in health facilities, whether it's COVID or not. It's been a struggle for many years in the TB world to actually get mask wearing normalized and so mask wearing for anybody who's coughing at any time wherever you are and then mask wearing in health facilities. And also enclosed spaces like you know, taxis etc. The difficulty will be sustaining that long term, but other countries like China have done that. If you place a spotlight, you can get the whole country behind one thing. The whole country has never been behind any other health issue.' Manager, March 2022, WC
